# A metabolomic strategy defines the regulation of lipid content and global metabolism by Δ9 desaturases in *Caenorhabditis elegans*

**DOI:** 10.1186/1471-2164-13-36

**Published:** 2012-01-20

**Authors:** Cecilia Castro, Funda Sar, W Robert Shaw, Masanori Mishima, Eric A Miska, Julian L Griffin

**Affiliations:** 1 Department of Biochemistry, University of Cambridge, 80 Tennis Court Road, Cambridge, CB2 1GA, UK; 2 Cambridge Systems Biology Centre, University of Cambridge, 80 Tennis Court Road, Cambridge, CB2 1GA, UK; 3 Wellcome Trust Cancer Research UK Gurdon Institute, The Henry Wellcome Building of Cancer and Developmental Biology, University of Cambridge, Tennis Court Road, Cambridge, CB2 1QN, UK; 4 The Medical Research Council Human Nutrition Research, Elsie Widdowson Laboratory, Fulborn Road, Cambridge, CB1 9NL, UK

## Abstract

**Background:**

*Caenorhabditis elegans *provides a genetically tractable model organism to investigate the network of genes involved in fat metabolism and how regulation is perturbed to produce the complex phenotype of obesity. *C. elegans *possess the full range of desaturases, including the Δ9 desaturases expressed by *fat-5, fat-6 *and *fat-7*. They regulate the biosynthesis of monounsaturated fatty acids, used for the synthesis of lipids including phospholipids, triglycerides and cholesteryl esters.

**Results:**

Liquid chromatography mass spectrometry (LC-MS), gas chromatography mass spectrometry (GC-MS) and nuclear magnetic resonance (NMR) spectroscopy were used to define the metabolome of all the possible knock-outs for the Δ9 desaturases, including for the first time intact lipids. Despite the genes having similar enzymatic roles, excellent discrimination was achievable for all single and viable double mutants highlighting the distinctive roles of *fat-6 *and *fat-7*, both expressing steroyl-CoA desaturases. The metabolomic changes extend to aqueous metabolites demonstrating the influence Δ9 desaturases have on regulating global metabolism and highlighting how comprehensive metabolomics is more discriminatory than classically used dyes for fat staining.

**Conclusions:**

The propagation of metabolic changes across the network of metabolism demonstrates that modification of the Δ9 desaturases places C.elegans into a catabolic state compared with wildtype controls.

## Background

Regulatory networks that govern fat and glucose metabolism are optimized to expend carbohydrates and accumulate fat when food intake is abundant, and switch to the consumption of stored fat when food is scarce. Even subtle deregulation of these pathways can bring about obesity [1,2], a significant risk factor for major diseases including hypertension, diabetes, coronary heart disease, myocardial infarction, liver disease and some cancers [3]. While obesity is a multifactorial feature, studies on twin and adopted children susceptibility to fat accumulation demonstrate that genetic predisposition is a key contributing factor in obesity [4-9]. Thus, it is important to understand how the network of genes involved in fat metabolism exerts regulation across the whole system to regulate fat synthesis and storage.

The nematode *Caenorhabditis elegans *has become a popular model for exploring the genetic basis for the regulation of fatty acid synthesis and storage [10]. Although worm and mammalian physiologies differ greatly, many of the proteins involved in synthesizing, oxidising and transporting fats, as well as many of the fat-regulatory components are highly conserved between *C. elegans *and mammals [11]. Furthermore, the genetic tractability of *C. elegans *allows one to take a global perspective for a complex trait such as the ability to store fat. A systematic RNAi screen of the *C. elegans *genome identified 305 genes associated with reduced body fat and 112 gene associated with increased fat storage [12] providing a powerful tool for the modelling of fat metabolism at the whole organism level. Because of the complexity of metabolic regulation, a genetically tractable system like *C. elegans *offers exceptional potential to unravel the connections across the genome that regulate fat metabolism and have led to the discovery of mammalian genes involved in energy balance [13,14].

*C. elegans *expresses the full range of desaturases activities found in plants (Δ12 and ω3 desaturase) and animals (Δ5, Δ6 and Δ9 desaturase) [15]. The Δ9 desaturases are the rate limiting enzymes in the biosynthesis of monounsaturated fatty acids which are used as preferred substrates for the synthesis of various kinds of lipids including phospholipids, triglycerides and cholesteryl esters. The crucial role of these enzymes arises because unsaturation of a fatty acid chain is a major determinant of the melting temperature of triglycerides, as well as the fluidity of biological membranes [16]. As a key control point in metabolic regulation, Δ9 has been proposed as a therapeutic target for the treatment of obesity, diabetes, and cardiovascular disease [17].

In *C. elegans*, the *fat-5 *gene encodes a palmitoyl-CoA desaturase, while the *fat-6 *and *fat-7 *genes encode stearoyl-CoA desaturases, and gene knockout strains have been generated and characterised using phenotypic analysis and gene expression, the vital staining of fats, the profiling of total fatty acids by gas chromatography (but not the profiling of intact lipids) [15,18,19]. While the single mutants displayed no obvious abnormal phenotypes, likely due to compensation by the other desaturases, the double mutants showed slow growth and reduced viability at low temperature, and the triple mutant was lethal unless supplemented with dietary oleic acid [18,19].

However, while providing an important insight into the consequences of the deletion on the organism, such approaches have only partially described the variations in the global metabolism, focusing on changes in the composition of fatty acids, and not how these changes interact with other metabolic pathways, or how they influence the composition of complex lipids found within an organism. These aspects are crucial to be studied due to the central role of the Δ9 enzyme and its products in the regulation of metabolism. Furthermore, questions have been raised as to which lipid species are stained by commonly used fat stains in genome wide screens of fat metabolism. In this study we have investigated the impact of deletion of *fat-5, fat-6 *and *fat-7 *on both global systemic metabolism and, for the first time, intact lipids by LC-MS. Using a range of techniques, including gas chromatography mass spectrometry (GC-MS) and nuclear magnetic resonance (NMR) spectroscopy in addition to LC-MS, we aim to demonstrate that these deletions show marked changes in lipid composition and place *C. elegans *in a catabolic state not just in lipid metabolism but also in terms of the impact on the TCA cycle and amino acid metabolism, demonstrating the influence the Δ9 desaturases have on regulating global metabolism.

## Results

To understand the consequences of Δ9 desaturase deletion on the metabolism of the whole organism in *C. elegans*, mutants defective in this enzyme were analysed using a wide range of metabolomic techniques, that allowed us to characterize both aqueous and lipid metabolites. We complemented our analysis by visualizing the fat content by vital and fixative staining methods. The aim is to integrate the rapid but generic results of the dyes with the deeper insights into metabolic changes obtained using the NMR or LC-MS techniques. As already reported in previous publications [19,20] at 20°C the *fat-6;fat-7 *double mutant animals display a greatly reduced fertility and slow growth. These characteristics made the strain unsuitable for our studies, as it was neither possible to generate a sufficient number of animals in parallel with the other strains to be analysed, nor separate the metabolic changes associated with the genetic modifications per se and the restriction in growth/development.

### Staining of lipid deposits

We hypothesised that animals partially lacking Δ9 desaturase activity may have altered lipid content. To address this, global levels of fat storage in wild type and Δ9 desaturase mutants were assessed. The wild type, *fat-5, fat-6 *and *fat-7 *mutants, as well as *fat-5;fat-6 *and *fat-5;fat-7 *double mutants were stained with both the vital dye Nile Red and with the fixative dye Oil Red-O, both of which have previously been used to characterize fat storage in *C. elegans *[12,21].

**Figure 1:**
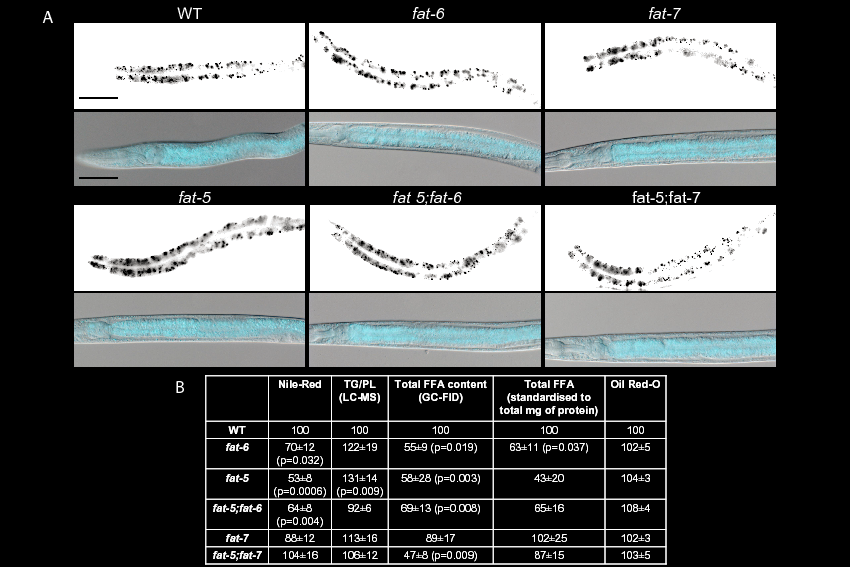
**Vital and fixative stainings of the Δ9 desaturase mutants**. (**A**) Representative images of the Nile Red-stained worms (upper rows for each strain) and of the Oil Red-O (lower rows) for the mutant and wild type strains. In each image, anterior is positioned to the left. Scale bar is 50 μm. (**B**) Quantification of the Nile-Red staining (n = 12 animals at least per genotype), of the Oil Red-O (n = 20 animals at least per genotype), of the global fatty acid content as measured by GC-FID (n = 8 replicates at least per genotype) and triglyceride/phospholipid measurements as obtained by LC-MS (n = 8 animals at least per genotype). The GC-FID data were normalised to the same number of worms in addition to the added internal standard (in the first column) and normalised for the internal standard and the total mg of protein, as measured by the Bradford assay for protein content (subsequent column). Data are reported as mean ± standard error of the mean (SEM).

Quantification of Nile Red staining in L4 animals showed significant decreases in the intensity of staining for *fat-5 *(p = 0.0006), *fat-6 *(p = 0.032) and *fat-5;fat-6 *(p = 0.004) genotypes (Figure 1A and 1B) compared with wild type. Using Nile red staining, as previously reported the *fat-6;fat-7 *mutant had a more pronounced reduction in lipid accumulation [19]. However, use of Nile Red has been criticized recently, suggesting that the dye could be treated as a xenobiotic by the animal and partitioned into a degradative compartment [22-24]. The Oil Red-O assay was performed (Figure 1A), but did not show any significant difference between the strains (Figure 1B).

The overall content of fatty acids (Figure 1B), as quantified by GC-FID, decreased in mutants significantly compared with wild type for all the strains except the *fat-7 *mutant. However, the ratio between triglycerides and phospholipids (Figure 1B), as obtained by LC-MS, showed no significant differences between wild type and any of the Δ9 desaturase mutant genotypes, except the *fat-5 *mutant: these results indicate that there is genetic redundancy between the Δ9 desaturases [18,19] and suggest more complex remodelling of the lipid fraction than a simple decrease in the triglyceride content. Therefore, these changes were investigated further in these mutants.

### NMR analysis of aqueous extracts

**Figure 2:**
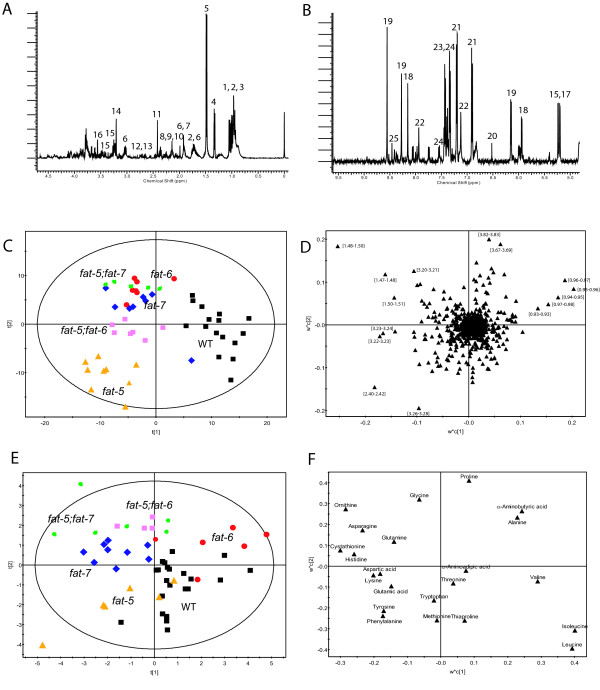
**Analysis of the aqueous phase metabolites in extract of *C.elegans***. ^1^H-NMR spectrum (A) between 0 and 5 ppm and (**B**) between 5 and 9.5 ppm for a selected illustrative sample. Key: 1. Ile; 2. Leu; 3. Val; 4. lactate; 5. Ala; 6. Lys; 7. acetate; 8. Glu; 9. Gln; 10. Met; 11. succinate; 12. Asp; 13. Asn; 14. choline; 15. glucose; 16. Gly; 17. trehalose; 18. GTP; 19. ATP; 20. fumarate; 21. Tyr; 22. His; 23. Phe; 24. Trp; 25 formate. (**C**) Score plot showing the clustering pattern according to genotype in the metabolic profiles obtained by NMR spectroscopy. Results from PLS-DA following the MSCA filter on the NMR spectra of all strains. The black squares represent samples belonging to the wild type strain, the red dots samples belonging to *fat-6 *mutant, the blue diamonds *fat-7 *mutant, the green stars *fat-5;fat-7 *mutant, the orange triangles *fat-5 *mutant and the pink squares *fat-5;fat-6 *mutant. (**D**) Loading plot for the score plot in **C**, shows the metabolites responsible for the discrimination among the groups: results from PLS-DA following MSCA filter on the NMR spectra of all strains. (**E**) Score plot of PLS-DA following MSCA filter on the amino acids analysis of all strains using the Phenomenex EZFaast kit. For the key to symbols see above. (**F**). Loading plot of PLS-DA following MSCA filter on the amino acids analysis of all strains.

Proton-NMR spectroscopy was used to profile a range of aqueous metabolites including amino acids, organic acids, sugars and metabolites associated with lipid metabolism (Figure 2A, B and Additional file 1: Table S1). Principal components analysis (PCA) was performed on the NMR spectra of all the strains to characterise the major changes in the metabolic profiles (data not shown; 12 PCs, R^2 ^= 85%, Q^2 ^= 54%). However, the first PC (explaining ~30% of the total variance) revealed differences between batches of animals. To highlight the metabolic variation due to the Δ9 desaturase knock-out, Multilevel Simultaneous Component Analysis (MSCA) was applied to the data set. The Partial Least Squares-Discriminate Analysis (PLS-DA) model obtained from the within-batches term of the MSC analysis has 6 latent variables (by cross-validation, with R^2^(X) = 56%, R^2^(Y) = 64%, Q^2 ^= 34%) and separated the mutants according to genotype (Figure 2C). The co-clustering between the metabolic profiles of *fat-6 *(BX106) and *fat-7 *(BX153) mutants may be expected as both *fat-6 *and *fat-7 *encode stearoyl-CoA desaturases and they were shown to have functional redundancy [18]. Clear discrimination was achieved for the two double mutant strains: *fat-5;fat-7 *(BX160) has a metabolic profile similar to the single mutants, while *fat-5;fat-6 *(BX110) lies in-between the two single mutants.

Analysis of the loadings plot was used to identify the metabolites most responsible for this discrimination (Figure 2D) - succinate (δ 2.42; signifying resonance identified), alanine (δ 1.48), choline (δ 3.20) and glycerophosphocholine (δ 3.26) had increased concentrations in mutant strains, while branched chain amino acids (δ 0.96-1.05) were increased in the wild type.

### Amino Acid analysis of the aqueous extracts

A targeted analysis of amino acids was performed by GC-MS [25], identifying 22 amino acids (Additional file 1: Table S2). As for the NMR experiments, a MSC filter was applied to the amino acid data set, to remove the variability due to the batches. The PLS-DA model obtained from the within-batches term of the MSC analysis had 5 latent variables by cross validation with a R^2^(X) = 63%, R^2^(Y) = 59% and a Q^2 ^= 32% (Figure 2E). This model produced good separation according to genotype including a notable distinction between the two single mutants lacking one of the two stearoyl-CoA desaturases, *fat-6 *and *fat-7*.

The loading plots associated with this analysis (Figure 2F) demonstrated an increased concentration of branched chain amino acids (leucine, isoleucine and valine) was found in the wildtype, while in the mutants there is a higher content of ornithine, cystathionine, asparagine and lysine. The *fat-6 *mutant was categorized by a particularly high concentration of alanine. Additional file 1: Table S3 summarises the characteristics of all the models built considering each mutant strain against the wild type. The list of the most significant amino acids changes considering each mutant against the wild type is shown in Additional file 1: Table S4.

The lipid phase was characterised both by GC-FID and LC-MS. The first technique allows a characterization of the fatty acids present in the samples, without considering the origin of the fats as they are cleaved from the original lipid species during the trans-esterification. Thus, LC-MS was also used to profile the intact lipids.

### GC-FID analysis of lipid extracts

Using GC-FID analysis of the *C. elegans *lipid extracts it was possible to identify straight chain fatty acids, branched chain fatty acids and cyclopropane fatty acids (Additional file 1: Table S5). As already reported [18,26], the nematode is able to synthesize *de novo *branched chain fatty acids from branched chain-CoA derived from leucine, valine and isoleucine. The cyclopropane fatty acids are synthesized by *E. coli *and absorbed through the diet.

In Table 1, the ratios between selected fatty acids highlighting the activities of the desaturase enzymes are reported. Considering the single mutants, the ratio between C16:1 and C16:0 was decreased for the *fat-5 *mutant, as expected due to the fact that the palmitoyl-CoA desaturase has been knocked out; the ratio between C18:1n9 and C18:0 decreased for both *fat-6 *and *fat-7 *mutants, as expected for the reduction in expression of stearoyl-CoA desaturase. However, in the *fat-5 *mutant the ratio C18:1n9/C18:0 is significantly increased compared with the wild type (p = 8.76 *10^-6^), suggesting an increased production of C18:1n9 to compensate for the lack of C16:1 and C18:1n7, the most abundant monounsaturated fatty acids. The ratios between C20:4n6 and C20:3n6, in both *fat-6 *and in *fat-7 *mutants, and between C20:3n6 and C18:2n6, in *fat-5 *mutants, were also changed as would be expected following the reduction in concentration of one of the main precursors in the synthesis of polyunsaturated fatty acids. For the double mutants, surprisingly, the ratios were not altered in the majority of cases compared to the wild type. This suggests a more complex reorganization of the system due to the lack of the palmitoyl-CoA desaturase and one of the stearoyl-CoA desaturase.

**Table 1:** Relative changes of the desaturase indexes in the fat mutant strains.

	*fat-6*	*fat-5*	*fat-5;fat-6*	*fat-7*	fat-5;fat-7
SCD-16 (16:1/16:0) (fat-5)	0.5 ± 0.1	0.33 ± 0.06 (p = 0.000156)	0.8 ± 0.1	0.4 ± 0.1	0.12 ± 0.03 (p = 0.039839)
SCD-18 (18:1/18:0) (fat-6, fat-7)	0.46 ± 0.07 (p = 0.011178)	0.15 ± 0.05 (p = 8.7E-06)	0.9 ± 0.4	0.55 ± 0.09 (p = 0.063)	1.1 ± 0.2
D5D (20:4(n6)/20:3(n6)) (fat-4)	1. 2 ± 0.1 (p = 0.000076)	0.8 ± 0.1	1.01 ± 0.06	1.2 ± 0.1 (p = 0.00028)	1.1 ± 0.1 (p = 0.0015)
D6D (20:3(n6)/18:2(n6)) (fat-3)	1.1 ± 0.1	0.91 ± 0.08 (p = 0.031)	0.9 ± 0.2	0.9 ± 0.1	0.86 ± 0.08 (p = 0.049)

SCD-16 is the Steroyl-CoA Desaturase (Δ9) specific in converting palmitic acid to palmitoleic; SCD-18 is the Steroyl-CoA Desaturase (Δ9) specific in converting stearic acid in oleic; D5D is the Δ5 desaturase; D6D is the Δ6 desaturase. In parenthesis the gene expressing the enzyme is indicated. Data are reported as ratio between the ratio of the mutant and the one of the wild type, in parenthesis t-test results for significant changes are shown.

**Figure 3:**
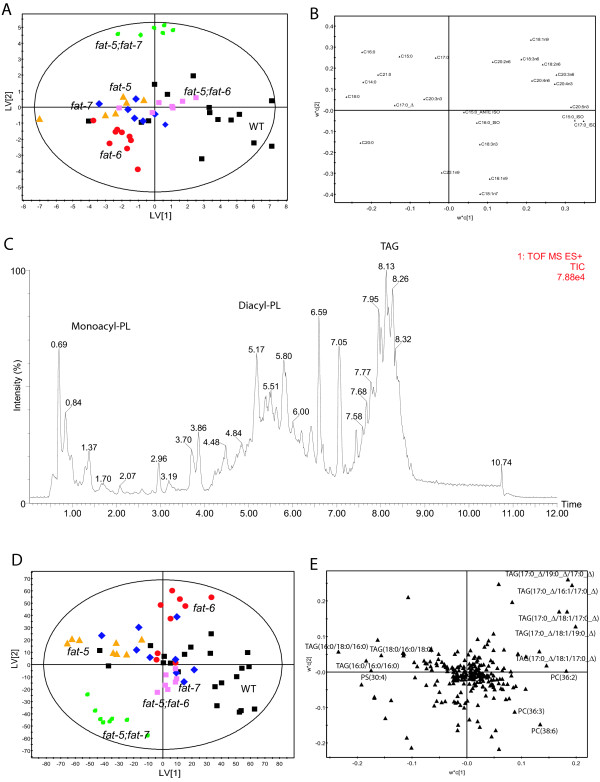
**Analysis of the lipid phase of the extracts**. (**A**) Score plot of PLS-DA following MSCA filter on the total fatty acid content as measured by GC-FID analysis of all strains. For the meaning of the symbols see key in Figure 2C. (**B**) Loading plot of PLS-DA model in **A**. (**C**) Lipid chromatogram for a selected illustrative sample of the lipid fraction obtained by reversed phase Ultra Performance Liquid Chromatography coupled to high resolution Mass Spectrometry (positive ion mode). Key: PL: PhosphoLipids; TAG: Triacylglycerols. (**D**) Score plot of PLS-DA following MSCA filter on the UPLC-MS experiments on the intact lipid phases of all strains. For the meaning of the symbols see the key in Figure 2C. (**E**). The loading plot of the PLS-DA model in D.

PLS-DA was applied to the within-batches term from MSCA of the GC-FID total fatty acids (FA) data across all of the strains (Figure 3A; 5 latent variables by cross validation, with R^2 ^X = 76%, R^2^Y = 58% and Q^2 ^= 45%). This model demonstrated good separation among all the strains: the double mutant *fat-5*;*fat-7 *had the most different profile. In addition the two single mutants lacking one of the stearoyl-CoA desaturase genes showed differences in their fatty acid content.

From an examination of the loadings plot (Figure 3B) mutant strains had higher concentrations of saturated FAs, reflecting the reduced capacity to convert them into monounsaturated FAs. There was a general decrease in the intensities of the PUFAs. There was also a decrease in the branched fatty acids to an extent that the ratio between total content of PUFA and branched fatty acid (BFA) increased markedly from the wild type to the mutant strains (PUFA/BFA = 5 ± 4 for wild type, 4.5 ± 0.8 for *fat-*5, 13 ± 3 for *fat-6 *mutant (p = 0.021), 11 ± 4 for *fat-7 *(p = 0.05), 13 ± 1 for *fat-5;fat-7 *(p = 0.029) and 8.4 ± 0.6 for *fat-5;fat-6 *(p = 0.0031)).

For both the double mutant *fat-5;fat-6 *and wild type there was relatively more C18:1n9 compared with the other genotypes (Additional file 1: Table S6 displays the characteristics of the PLS-DA models considering each mutant against the wild type, while Additional file 1: Table S7 shows a list of the most significant changes in fatty acids considering each mutant compared with the wild type). The *fat-5;fat-6 *mutant strain produces no *fat-5*, so it cannot convert palmitic acid to palmitoleic (the intensity of palmitoleic was very low in this mutant) and thus, monounsaturated fats must be produced by *fat-7*, the only desaturase that is still functional. In the other double mutant (*fat-5;fat-7*) the absence of FAT-7 disrupts the normal ratio between oleic and stearic acid, despite retaining a functional copy of the highly expressed gene *fat-6*. This suggests there may be both redundant and non-redundant roles for FAT-6 and FAT-7.

### LC-MS analysis of lipid extracts

In Figure 3C, a typical chromatogram obtained analysing the intact lipids is shown. Again a MSCA/PLS-DA approach was used to produce a model that separated all the strains (Figure 3D; R^2 ^X = 49%, R^2^Y = 68% and Q^2 ^= 30%). This model passed cross validation and more robust models were produced when two-way comparisons were considered with respect to the wild type strain (Additional file 1: Table S8). In particular the two single mutants lacking one of the two stearoyl-CoA desaturase genes (*fat-6 *and *fat-7*) were characterised by very different lipid profiles and contributions to the loadings plots (Figure 3E). A common feature of most of the mutants, and in particular the single mutant *fat-6*, was the higher content of triglycerides containing fatty acids absorbed from the diet (in particular, TAG(17:0Δ/19:0Δ/17:0Δ); TAG(17:0Δ/16:1/17:0Δ); TAG(17:0Δ/18:1/17:0Δ); TAG(17:0Δ/18:1/19:0Δ)), while others (in particular the single mutant *fat-5 *and the double mutant *fat-5;fat-7*) had a higher content of triglycerides containing saturated fatty acids (in particular, TAG(18:0/16:0/18:0); TAG(16:0/18:0/16:0); TAG(16:0/16:0/16:0)). Furthermore, decreased content of phosphocholine-lipids containing unsaturated fatty acids (PC(36:3); PC(38:6)) was found in the mutants compared with the wild type strain.

### Gaussian Graphical Models on GC-FID Experiments of the lipid fraction

The results obtained by the GC-FID experiments of the lipid fraction were also analysed using Gaussian Graphical Models (GGM), weighted networks where nodes, representing the measured entities (fatty acids in this case), are connected through edges annotated with the partial correlation coefficient between the two. The method aims to identify the correlations that remain between nodes if the effect of all the other variables has been removed from the contribution.

**Figure 4:**
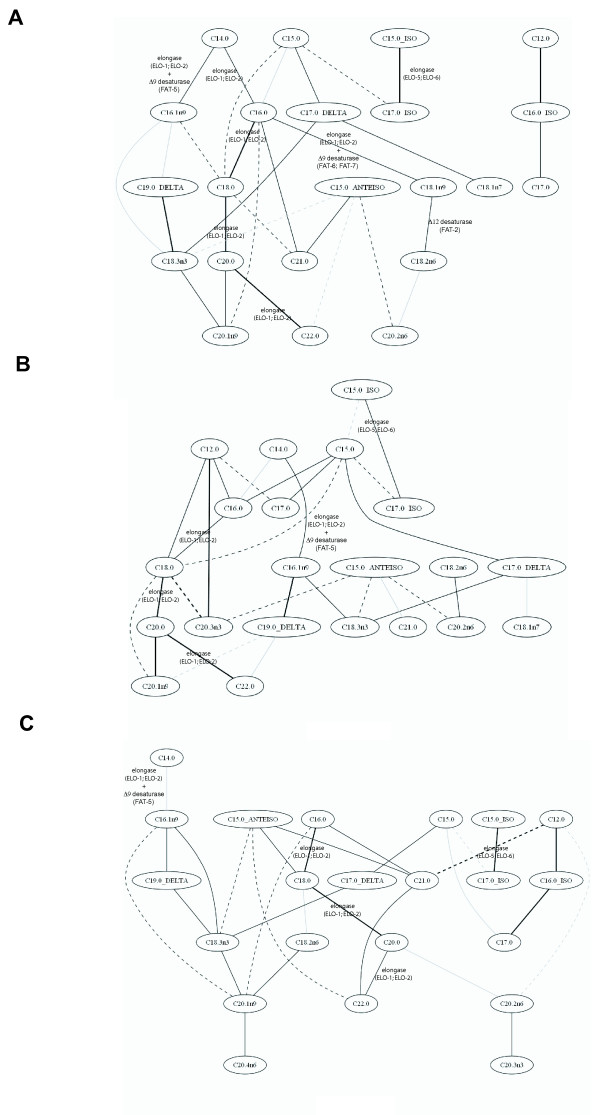
**Graphical Gaussian Model obtained considering the 30 highest partial correlation coefficients from the results of the GC-MS experiments on the lipid fraction to measure total fatty acid content**. (**A**) The network calculated considering the fatty acid concentrations in all the strains. The network was also calculated considering wild type and *fat-6 *mutant (**B**) alone, and wild type and *fat-7 *mutant (**C**) alone. The straight lines represent positive partial correlation coefficients between the fatty acids, while dotted lines represent negative partial correlation coefficients. The intensity of the line is proportional to the intensity of the correlation.

In Figure 4A, the network obtained considering the 30 highest partial correlation coefficients of all the strains together is shown. The backbone of the synthesis of the straight chain fatty acids, from C14:0 to C22:0 is evident. Interestingly, there are branches from C14:0 and C16:0, linking either the next saturated fatty acid (catalytically through elongase) or to the next monounsaturated fatty acid (catalytically through elongase and Δ9 desaturase). Other strong partial correlations are between C15:0-ISO and C17:0-ISO (through the selective enzyme ELO-6) and C17:0-Delta and C19:0-Delta with C18:3n3 (both the cyclopropane fatty acids detected come from the diet).

In Figure 4B and 4C the network obtained considering the 30 highest partial correlation coefficients for each strain against the wild type are shown. In each case, the backbone of the fatty acid elongation pathway can be identified. It is possible to note certain correlation patterns associated with the deletion of a given gene - for example, the cluster formed by C15:0-ISO, C17:0-ISO and C18:1n7, in *fat-5 *mutants: C18:1n7 is synthesised by the elongation of C16:1n9, obtained by desaturation from C16:0 by FAT-5 palmitoyl-CoA desaturase. The correlation structure for *fat-6 *and *fat-7 *mutants are markedly different, even though the genes both encode a stearoyl-CoA desaturase, demonstrating their very different roles in regulating fat metabolism.

## Discussion

To date most studies focused on metabolic changes induced by modifying the expression of desaturases in *C. elegans *have used GC-based approaches to profile the total fatty acids present. In this study we extended this analysis to also include aqueous metabolism and intact lipids to demonstrate a profound perturbation in global metabolism induced by perturbing the expression of the desaturases. The perturbations detected across the network of metabolism highlights the usefulness of global profiling tools for functional genomics. The use of LC-MS to profile intact lipids provides greater detail in terms of the fatty acid changes when compared with GC alone, and the profiling of amino acid analysis provides a further insight into core aqueous metabolism, something which is often ignored in many studies of fat metabolism in *C.elegans*.

The expression profiles of stearoyl-CoA desaturases are regulated by a large number of transcriptional and environmental inputs. In turn, the activities of these enzymes affect a large number of processes in the organism. The direct effects of knocking-out a desaturase enzyme is to modulate the ratio of saturated to unsaturated fatty acids, but this in turns affects membrane fluidity, activates specific signalling pathways, and influences mobility or activity of membrane bound proteins. As part of this, reorganisation may happen through feedback mechanisms involving metabolites as signals, and hence a global metabolomic strategy is needed in order to fully define all of the perturbations induced across the network of metabolism and not only those in fatty acid synthesis.

The higher content of fatty acids in the wild type strain compared with the Δ9 desaturase mutants was demonstrated by both GC-FID and LC-MS analysis, yet it was not detected by Oil Red-O staining. Previously, the fat content of these mutant strains has been measured by coherent anti-Stokes Raman scattering (CARS) [27], showing a 1.4-fold decrease in the expression level of neutral lipid droplets for the *fat-5;fat-6 *double mutant. One reason for this discrepancy between the Oil Red-O staining and other methods is that the Oil Red-O method is typically not sensitive enough to detect such a variation. The decrease detected by GC and LC-MS is not only due to a reduction in the actual synthesis of the lipids, but also due to an increase in the catabolism of the fatty acids, with dietary fatty acids representing a larger proportion of the total fatty acid compliment. This is also supported by the increase in succinate, which indicates an increase in acetyl-CoA metabolism via the glyoxylate cycle. A dramatic increase in the expression of genes involved in mitochondrial and peroxisomal β-oxidation has been reported in mutants where the desaturases have been knocked out [19]. In mice, the deletion of SCD-1 leads to a decrease in the content of TAG and cholesterol esters and down-regulates de novo fatty acid synthesis. Furthermore, the deletion reduces lipid content by enhancing oxidation [28] and thus, an increase in catabolism following reduced desaturase activity appears to be highly conserved. It has also been shown that during fasting the concentration of stearic acid is maintained due to a strong fasting-dependent repression of the *fat-7 *gene, when peroxisomal and mitochondrial fatty acid β-oxidation are selectively stimulated [29], suggesting a regulatory interaction between *fat-7 *and β-oxidation, possibly by sensing the concentration of stearic acid. Moreover, an increase in the fatty-acid-binding proteins *lpd-1*-*lpd-7 *has been observed in the desaturase knocked-out mutants [19]: these proteins sequester and transport fatty acids and acyl-CoA esters, facilitating their intracellular utilization, storage and signalling functions. An increase in their expression suggests an increment in the traffic of the fatty acids in the cell and is in agreement with an increase in their catabolism detected in this study.

However, this is not the only reorganization in the lipid content of the mutants. The results show a reduction in some of the phosphocholine derivatives in the mutants, in particular in those containing unsaturated fatty acids, suggesting a reorganisation of the cell membrane. This may be because the cell membranes have a reasonable amount of tolerance to the inclusion of saturated fatty acids, and the conservation of PUFAs for specific roles such as the synthesis of signalling molecules. Indeed, there is an enhancement in the heat-stress tolerance when animals are treated with RNAi for *fat-6 *and *fat-7 *[30] arising from the increased incorporation of saturated fatty acids in the membranes. The changes in phosphocholine metabolism could only be followed in this high level of detail by the use of LC-MS. The approach detects the lipids directly and using fragmentation patterns we can deduce how changes in total fatty acid profiles effect the different phospholipids found in *C.elegans*.

The efficient storage of lipids within lipid droplets appears to be highly dependent on the generation of MUFAs. Unsaturated FAs, but not saturated FAs, induce the formation of lipid droplets or increase the size of the existing lipid droplets. This is confirmed by the fact that when supplemented with vaccenic acid (18:1n7) the concentration of TAGs were increased in mutants with peroxisomal dysfunctions [31]. Furthermore, an impairment in desaturase activity is correlated with a decreased ability to generate and expand lipid droplets to facilitate TAG storage. Under conditions where MUFAs are in short supply to prevent the accumulation of toxic saturated lipids within the cytoplasm mitochondrial and peroxisomal β-oxidation is stimulated [19], possibly through the action of *nhr-49*, the *C. elegans *homologue of PPARα.

The fact that both BFAs and MUFAs decrease in the present study further suggests a general increase in the catabolism of fatty acids, and thus while unsaturated fats may compensate for a lack of BFAs the reverse is not necessarily true. Indeed, when the double mutant *fat-6;fat-7 *is treated with *nhr-64(RNAi) *(a gene, whose knock-down suppresses the low fat phenotype of the double mutant) an increase in the concentrations of branched fatty acid was found. This was attributed to a general increase in fatty acids synthesis [20].

There is an inverse correlation between the concentration of C17iso in TAGs and fat storage levels [24]. C17iso is proposed to act as a chemical/nutritional indicator of the metabolic state of *C. elegans*. As this fatty acid is the final step of a synthesis that is initiated from the amino acid leucine, the concentration of C17iso may reflect the concentrations of essential dietary amino acids and more generally protein and lipid homeostasis in the organism. Δ9 desaturase-deleted mutants have both reduced content of branched fatty acids and essential amino acids.

Previously, the focus of metabolic studies on these strains has been on the changes in the fatty acid composition. In the present study, in addition to profiling intact lipid metabolites and cleaved fatty acids, also a non-targeted profiling of the aqueous phases was performed, demonstrating that the changes associated with the deletion of the desaturases are not limited to the lipid fractions but extend to the entire metabolome. The increase in the concentration of alanine in the mutants, indicative of increased glycolysis and consumption of amino acids, as well as an increase in succinate suggesting increased metabolism through the citric acid cycle are indicative of a general increase in catabolism. Furthermore, the increased content of branched amino acids in the wild type compared to the mutants seems to confirm this higher catabolic state accompanying the deletions of the Δ9 desaturase genes. These amino acids are the starting point for the synthesis of the branched fatty acids, but both branched chain amino acids and fatty acids decrease in concentration in the mutant, suggesting increased catabolism of both metabolite classes. The higher content of choline, a precursor of membrane phospholipids, in the mutants compared to wild type is another indicator of the lipid reorganization.

There is numerous evidence suggesting that *fat-6 *and *fat-7 *have both redundant and non-redundant functions. Our results show differences in the behaviour of both the two single mutants and the two double mutants. The analysis of the amino acid profiles by GC-MS indicates a different influence of the two genes on other metabolic pathways, such as amino acid anabolism and catabolism. The analysis on the intact lipids also demonstrated an increase in absorption of cyclopropane fatty acids in the *fat-6 *single mutant to compensate the absence of desaturation. This strain lacks the most highly expressed Δ9 desaturase. Other strains, instead, show an increase in the content of saturated fatty acids in the lipids. These results are confirmed also by the analysis of fatty acids by GC-FID: there is an increase in the content of C17:0_Δ in the *fat-6 *verses wild type model, not present in the *fat-7 *verses wild type model. However, without LC-MS it would have not been possible to follow this remodelling of the triglycerides.

## Conclusions

We have shown that deletion of Δ9 desaturases have profound effects on metabolism in *C. elegans *that far extend from the initial reduction in MUFAs. In particular, the major results obtained by our comprehensive characterization were that:

i. the mutants had increased accumulation of triglycerides from dietary or saturated fats. However, overall the total fat content was decreased in the mutants compared to the wild type indicative of increased catabolism;

ii. a reduction in some phosphocholine derivatives in the mutants, especially those containing unsaturated fatty acids, suggest reorganisation of cell membranes.

iii. a general increase in fatty acid and amino acid catabolism in the mutants.

Currently the majority of studies of fat storage in *C. elegans *rely on whole animal staining, gas chromatography analysis of total fatty acids or thin layer chromatography of lipid classes. However, the propagation of the changes into a wide range of metabolic pathways highlighting the usefulness of global profiling tools for functional genomics.

## Methods

### Culture of nematodes

*C. elegans *were grown under standard conditions on Nematode Growth Media (NGM) with HB101 [32] as food source, at 20°C. The wild-type strain used was Bristol N2 [33]. Mutant strains were obtained from the Caenorhabditis Genetics Center, University of Minnesota, Twin Cities, MN and included: BX107 *fat-5(tm420) V*; BX106 *fat-6(tm331) IV*; BX153 *fat-7(wa36) V*; BX110 *fat-7(wa36) V fat-5(tm420) V *(referred to as *fat-5;fat-7 *in the text); BX160 *fat-6(tm331) IV;fat-5(tm420) V *refered to as *fat-5;fat-6*); and BX156 *fat-6(tm331) IV;fat-7(wa36) V*. Mutations were verified using PCR and DNA sequencing prior to starting experiments (details of primers available on request). For each experiment, early embryos were isolated by alkaline hypochlorite treatment [33] and plated after synchronisation on NGM plates seeded with bacteria. For each biological replicate, ~8000 animals were harvested, washed and stored at -80°C until extraction. Eight samples were grown for each strain.

### Nile Red and Oil Red-O staining and quantification

Nile Red staining was performed as described previously [12]. Images were acquired on an Axio Imager A1 Microscope (Zeiss) fitted with a CCD camera (Hamamatsu Orca ER 12 bit) and OpenLab(Version 4.0.4) programme, using identical settings and exposure times to allow direct comparisons. Reported Nile Red intensities were quantified using ImageJ software (NIH) on 8-10 animals of each genotype randomly selected. Each experiment was repeated at least three times.

Oil Red-O staining was performed as described previously [21]. Briefly, young adult animals were washed from plates into 120 μL of PBS, pH8, to which an equal volume of 2× MRWB buffer containing 2% paraformaldehyde was added. Animals were taken through three freeze-thaw cycles between a dry-ice/ethanol bath and warm running tap water and fixed for 1 hour at 4°C. They were then washed in PBS to remove PFA, and incubated in 60% isopropanol to dehydrate, and then in a 60% Oil Red-O stain solution. Images were acquired on an Axio Imager A1 Microscope fitted with a QICAM 1394 CCD camera using identical settings to keep the background illumination constant across different samples.

For the quantification, the image was imported in ImageJ and thresholded to identify the worm area. In the original image, the averaged intensity values of the green and blue channels were subtracted from the red channel, and the total intensity calculated subtracting the mean intensity of background from the mean intensity across the picture. The obtained value was normalized by the area of the animals within the field of view of the image.

### Extraction procedure for metabolites

Metabolites from whole nematodes were extracted using a methanol-chloroform procedure. 600 μl of methanol-chloroform (2:1 v:v) was added to the frozen nematodes and samples were sonicated for 15 min. 200 μl each of chloroform and water were added, the samples centrifuged and the aqueous layer separated from the lipid one. The procedure was repeated twice. The aqueous layer was dried overnight in an evacuated centrifuge, while the lipid fraction was dried overnight in the fumehood.

### Analysis of aqueous extracts

*NMR spectroscopy*: The dried extracts were rehydrated in 550 μl D_2_O, containing 0.05 mM (sodium-3-(tri-methylsilyl)-2,2,3,3-tetradeuteriopropionate (TSP) (Cambridge Isotope Laboratories, MA, USA) as an internal standard. The samples were analysed using an AVANCE II+ NMR spectrometer operating at 500.13 MHz for the^1^H frequency (Bruker, Germany) using a 5 mm TXI probe. Spectra were collected using a solvent suppression pulse sequence based on a one-dimensional NOESY pulse sequence to saturate the residual^1^H water signal (relaxation delay = 2 s, t_1 _increment = 3 us, mixing time = 150 ms, solvent presaturation applied during the relaxation time and the mixing time). One hundred and ninety-six transients were collected into 16 K data points over a spectral width of 12 ppm at 27°C. In addition, representative samples of nematodes were also examined by two-dimensional spectroscopy, including COSY (COrrelation SpectroscopY), in conjunction with reference to previous literature and databases [34-36] and the Chenomx spectral database contained in Chenomix NMR Suite 5.0 (Chenomx, Alberta, Canada)) for spectral assignment.

*GC-MS*: The samples analysed by NMR were dried overnight in an evacuated centrifuge and rehydrated in 150 μl of water. 100 μl were used for the analysis by the EZ:faast Free (Physiological) Amino Acid Analysis by GC-MS kit (Phenomenex, USA). The samples were analysed according to the procedure described by the manufacturer.

### Analysis of lipid extracts

Lipid extracts were dissolved in 200 μl of chloroform/methanol (1:1 v/v): half was used for GC-FID analysis of total fatty acids and half for LC-MS analysis of intact lipids. For the GC-FID analysis 50 μl of D-25 tridecanoic acid (200 μM in chloroform), 650 μl of chloroform/methanol (1:1 v/v) and 250 μl BF_3_/methanol (Sigma-Aldrich) was added to the extract and the vials were incubated at 80°C for 90 min. 500 μl H_2_O and 1 ml hexane were added and each vial vortex mixed. The organic layer was evaporated to dryness before reconstitution in 100 μl hexane for analysis. The derivatised organic metabolites were injected in a Focus GC and the column eluent was introduced into a flame-ionization detector (FID, Thermo Electron Corporation). The column used was ZB-WAX (Phenomenex; 30 m × 0.25 mm ID × 0.25 μm; 100% polyethylene glycol). The initial column temperature was 60°C and was held for 2 min. This was increased by 15°C min^_1 ^to 150°C then increased at a rate of 3°C min^_1 ^to 230°C. This final temperature was held for 10 minutes. Peaks were assigned using Food Industry FAME Mix (Restek 6098) and Bacterial Acid Methyl Ester (BAME) Mix solution (Supelco 47080).

For LC-MS 5 μl of each sample was analysed on a Waters Q-Tof Ultima mass spectrometer combined with an Acquity Ultra Performance LC (UPLC). The sample was injected onto a 1.7 μm bridged ethyl hybrid C8 column (2.1 × 100 mm; Waters Corporation) held at 65°C. The binary solvent system (flow rate 0.200 ml/min) included A. HPLC grade water (1% 1 M NH_4_Ac, 0.1% HCOOH) and B. LC/MS grade acetonitrile/isopropanol 5:2 (1% 1 M NH_4_Ac, 0.1% HCOOH). The gradient started from 65% A/35% B, reached 100% B in 6 min and remained there for the next 7 min. The data was collected over the mass range of m/z 100-1400 with a scan duration of 0.5 sec and an interscan delay of 0.1 s. The source temperature was set at 100°C and nitrogen was used as desolvation gas (600 L/h) at 300°C. The voltages of the sampling cone and capillary were 40 V and 3 kV, respectively and collision energy 5 V. Reserpine (50 μg/L) was used as the lock spray reference compound (10 μl/min; 10 sec scan frequency).

To assign the lipid species present, a representative sample for each strain was analysed by tandem mass spectrometry (MS/MS). MS/MS runs were performed using ESI+ mode and collision energies of 18, 20, 24, 30 V and a mass range of 80 to 1,100 m/z. Other conditions were as described above.

### Data processing

NMR spectra were processed using ACD one-dimensional NMR processor (vers. 8, ACD, Toronto, Canada). Free induction decays were Fourier transformed following multiplication by a line broadening of 1 Hz, and referenced to TSP at 0.0 ppm. Spectra were phased and baseline corrected manually. Each spectrum was integrated using 0.01 ppm integral regions between 0.5 and 4.5, and 5.5-9.5 ppm. Each spectral region was normalised to a total integral value of 1000. GC-FID chromatograms were analysed using Xcalibur, version 2.0 (Thermo Fisher), integrating each peak individually. Each integrated peak was normalised so that the total sum of peaks was set to 1000. LC-MS chromatograms were analysed using Micromass Markerlynx Applications Manager Version 4.1 (Waters Corporation). The ion intensities for each lipid identified were normalized so that the sum of the peak within each sample was set to 1000. Furthermore, the regions corresponding to the different classes of lipids were integrated from each chromatogram, to quantify them.

### Multivariate analysis of metabolic profiles

Each set of metabolic profiles obtained were analysed by multivariate analysis. Datasets were imported into SIMCA-P 11.0 (Umetrics, Umea˚, Sweden) for processing using PCA and PLS-DA (a regression extension of PCA used for supervised classification). ^1^H NMR data were Pareto scaled, in which each variable was centred and multiplied by 1/(S_k_)^1/2 ^where S_k _is the standard deviation of the variable. GC-MS data were scaled to unit variance by dividing each variable by 1/(S_k_). LC-MS data were Pareto scaled.

In addition, a Multilevel Simultaneous Component Analysis (MSCA) [37] was applied to the non-normalized data set. MSCA is a combination of Analysis-of-variance and Simultaneous Component Analysis and it enables analysis of metabolomic studies with an experimental design. It takes into account the multilevel structure in the data allowing a separate analysis and interpretation of the variation sources induced by the different factors in the experimental design. It was performed using Matlab (The MathWorks) with the routines available from van Velzen et al., 2008 http://www.bdagroup.nl/content/Downloads/software/software.php. The data set was split in a between-batches term (biological variation) and within-batches term (genetic variation). The batches were defined as follow: one contained N2 (wildtype), BX106 (*fat-6*), BX153 (*fat-7*) and BX160 (*fat-5;fat-7*); one N2 (wildtype) and BX107 (*fat-5*); and the third N2 (wildtype) and BX110 (*fat-5;fat-6*). The within-batches term was imported into SIMCA-P, Pareto (NMR and GC-FID data) or unit variance (GC-FID) scaled and analysed by PLS-DA.

### Gaussian Graphical Models of fatty acids analysed by GC-FID

The fatty acid peaks identified in the GC-FID chromatograms were normalised to the internal standard, imported into R and analysed by Gaussian Graphical Models (GGM), using GeneNet [[38]; http://strimmerlab.org/software/genenet/].

## Authors' contributions

JLG, EAM and CC conceived and designed the experiments. CC, WRS & FS performed the experiments and analysed the data. MM defined the quantification methods of the staining experiments. All authors wrote the paper. All authors read and approved the final manuscript.

## Supplementary Material

Additional file 1 **Supplemental Material. 1**. Figure S1 **2. Table S1**: List of the resonances assigned in the NMR spectra of *C. elegans*. **3. Table S2**. List of the amino acids identified by the EZ:faast Free (Physiological) Amino Acid kit in *C. elegans*. **4. Table S3**. Characteristics of the PLS-DA models obtained considering each strains against the wild type in the GC-MS experiments using the EZ:faast Free (Pysiological) Amino Acid kit. **5. Table S4**. List of the most significant amino acids changes considering each mutant against the wild type. **6. Table S5**. List of the fatty acids identified by the GC-FID analysis of *C. elegans*. **7. Table S6**. Characteristics of the PLS-DA models obtained considering each strains against the wild type in the GC-MS experiments of the lipid fraction. **8. Table S7**. List of the most significant fatty acid changes considering each mutant against the wild type. **9. Table S8**. Characteristics of each PLS-DA models obtained considering each strains against the wild type in the LC-MS experiments of the lipid fraction. Click here for file
