# Supplementary material for: A metabolomic strategy defines the regulation of lipid content and global metabolism by Δ9 desaturases in Caenorhabditis elegans
Source: BMC Genomics. 2012 Jan 20;13:36. doi: 10.1186/1471-2164-13-36 (PMC3398271; doi:10.1186/1471-2164-13-36)
Supplement: Additional file 1 — Supplemental Material. 1. Figure S1 2. Table S1: List of the resonances assigned in the NMR spectra of C. elegans. 3. Table S2. List of the amino acids identified by the EZ:faast Free (Physiological) Amino Acid kit in C. elegans. 4. Table S3. Characteristics of the PLS-DA models obtained considering each strains against the wild type in the GC-MS experiments using the EZ:faast Free (Pysiological) Amino Acid kit. 5. Table S4. List of the most significant amino acids changes considering each mutant against the wild type. 6. Table S5. List of the fatty acids identified by the GC-FID analysis of C. elegans. 7. Table S6. Characteristics of the PLS-DA models obtained considering each strains against the wild type in the GC-MS experiments of the lipid fraction. 8. Table S7. List of the most significant fatty acid changes considering each mutant against the wild type. 9. Table S8. Characteristics of each PLS-DA models obtained considering each strains against the wild type in the LC-MS experiments of the lipid fraction. [file 1471-2164-13-36-S1.DOC]

|  | **Description** | **pag.** |
| --- | --- | --- |
| **1.** | Figure S1 | **2** |
| **2.** | **Table SI**: List of the resonances assigned in the NMR spectra of *C. elegans*. | **3** |
| **3.** | **Table SII**. List of the amino acids identified by the EZ:faast Free (Physiological) Amino Acid kit in *C. elegans*. | **6** |
| **4.** | **Table SIII.** Characteristics of the PLS-DA models obtained considering each strains against the wild type in the GC-MS experiments using the EZ:faast Free (Pysiological) Amino Acid kit. | **7** |
| **5.** | **Table SIV**. List of the most significant amino acids changes considering each mutant against the wild type. | **8** |
| **6.** | **Table SV**. List of the fatty acids identified by the GC-FID analysis of *C. elegans*. | **9** |
| **7.** | **Table SVI.** Characteristics of the PLS-DA models obtained considering each strains against the wild type in the GC-MS experiments of the lipid fraction. | **10** |
| **8.** | **Table SVII**. List of the most significant fatty acid changes considering each mutant against the wild type. | **11** |
| **9.** | **Table SVIII.** Characteristics of each PLS-DA models obtained considering each strains against the wild type in the LC-MS experiments of the lipid fraction. | **12** |

**Supplementary Figure.**

**Figure S1.** Graphical Gaussian Model obtained considering the 30 highest partial correlation coefficients from the results of the GC-MS experiments on the lipid fraction to measure total fatty acid content. The network are calculated considering wild type and *fat-5* mutant (**A**) wild type and *fat-5;fat-6* mutant (**B**), wild type and *fat-5;fat-7* mutant (**C**) only. As in figure 4, the straight lines represent positive partial correlation coefficients between the fatty acids, while dotted lines represent negative partial correlation coefficients. The intensity of the line is proportional to the intensity of the correlation.


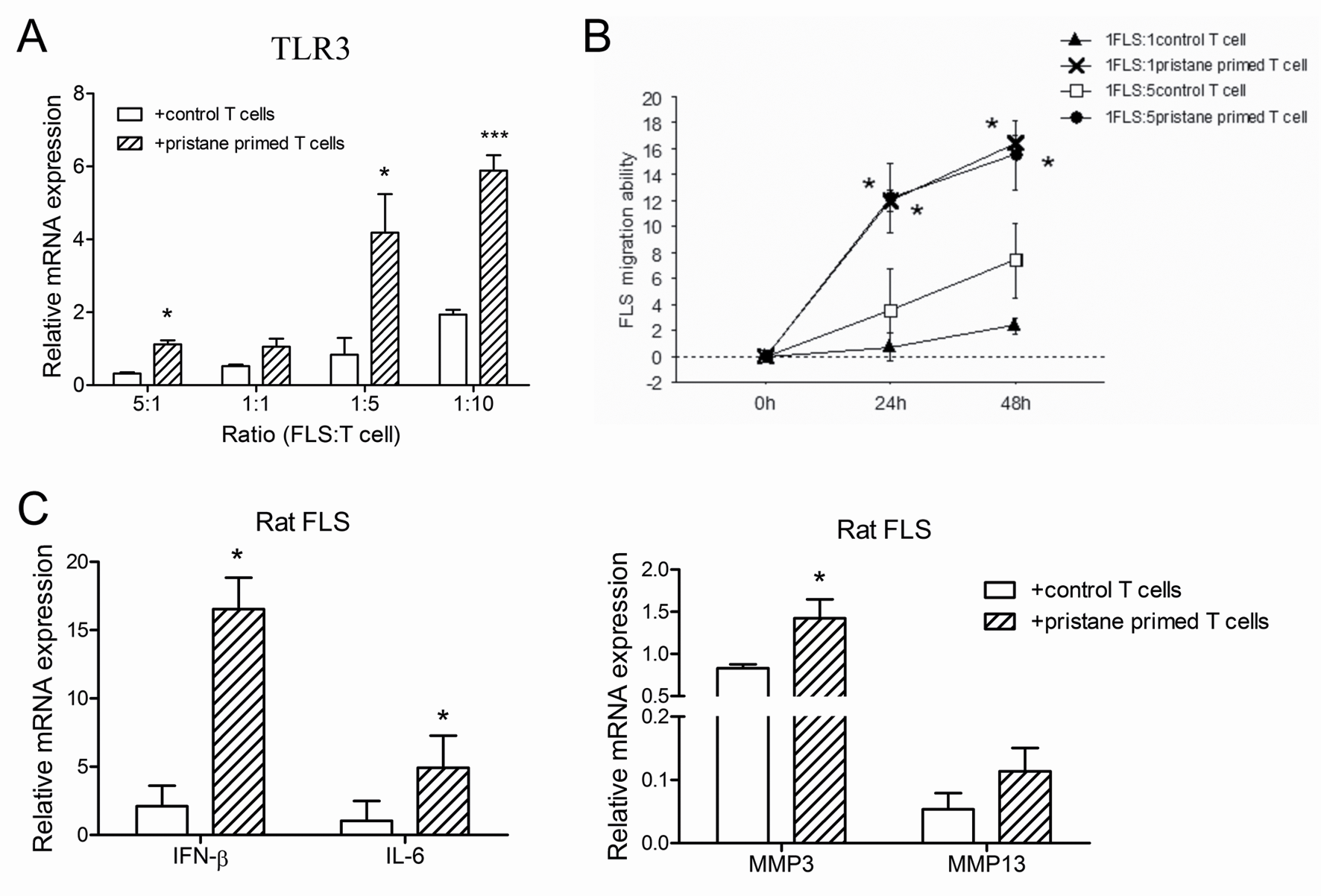


**Table SI**: List of the resonances assigned in the NMR spectra of *C. elegans*.

| ***(ppm)*** | ***Multiplicity*** | ***Metabolite*** |  |
| --- | --- | --- | --- |
| 0.93 | t | Ile | δ-CH_3_ |
| 0.94 | d | Leu | δ'-CH_3_ |
| 0.96 | d | Leu | δ-CH_3_ |
| 0.97 | d | Val | γ-CH_3_ |
| 0.99 | d | Ile | β-CH_3_ |
| 1.03 | d | Val | γ'-CH_3_ |
| 1.25 | m | Ile | γ-CH |
| 1.32 | d | Lactate | β-CH_3_ |
| 1.32 | d | Thr | γ-CH_3_ |
| 1.45 | m | Ile | γ'-CH |
| 1.49 | d | Ala | β-CH_3_ |
| 1.67 | m | Leu | γ-CH |
| 1.72 | m | Lys | δ-CH_2_ |
| 1.74 | m | Leu | β-CH_2_ |
| 1.89 | m | Lys | β-CH_2_ |
| 1.92 | s | Acetate |  |
| 1.93 | m | Ile | β-CH |
| 1.99 | s | Acetamide |  |
| 2.02 | m | Pro | γ-CH_2_ |
| 2.03 | m | Pro | β'-CH |
| 2.03 | m | Glu | β-CH |
| 2.12 | m | Glu | β'-CH |
| 2.13 | m | Cystathionine | C2H_2_ |
| 2.13 | m | Gln | β-CH_2_ |
| 2.14 | s | Met | S-CH_3_ |
| 2.19 | m | Met | β-CH_2_ |
| 2.26 | m | Val | β-CH |
| 2.33 | m | Pro | β-CH |
| 2.34 | dt | Glu | γ-CH_2_ |
| 2.42 | s | Succinate |  |
| 2.46 | m | Gln | γ-CH_2_ |
| 2.55 | t | β-Ala | α-CH_2_ |
| 2.63 | t | Met | γ-CH_2_ |
| 2.65 | dd | Asp | β-CH |
| 2.73 | m | Cystathionine | C4H_2_ |
| 2.8 | dd | Asp | β'-CH |
| 2.83 | dd | Asn | β-CH |
| 2.94 | dd | Asn | β'-CH |
| 3.03 | t | Lys | ε-CH_2_ |
| 3.06 | t | Ornithine | δ-CH_2_ |
| 3.11 | dd | Phe | β'-CH |
| 3.12 | m | Cystathionine | C1'H |
| 3.17 | t | β-Ala | β-CH_2_ |
| 3.2 | s | Choline | N(CH_3_)_3_ |
| 3.23 | t | β-Glucose | C2H |
| 3.28 | dd | Phe | β-CH |
| 3.33 | t | Pro | δ'-CH |
| 3.41 | dd | β-Glucose | C4H |
| 3.41 | t | Pro | δ-CH |
| 3.43 | dd | α-Glucose | C4H |
| 3.47 | dd | β-Glucose | C5H |
| 3.49 |  | β-Glucose | C3H |
| 3.5 | m | Choline | β-CH_2_ |
| 3.52 | t | α-Glucose | C2H |
| 3.54 | dd | Glycerol | C1, C3H |
| 3.56 | s | Gly | α-CH_2_ |
| 3.59 | d | Val | α-CH |
| 3.59 | d | Thr | α-CH |
| 3.64 | dd | Glycerol | C1, C3H' |
| 3.65 | d | Ile | α-CH |
| 3.72 | m | Leu | α-CH |
| 3.73 |  | α-Glucose | C3H |
| 3.74 | t | Lys | α-CH |
| 3.78 | q | Ala | α-CH |
| 3.81 | m | Cystathionine | C2H_2_ |
| 3.88 | dd | α-Glucose | C5H |
| 3.94 | m | Cystathionine | C2'H_2_ |
| 3.99 | dd | Phe | α-CH |
| 4.05 | m | Choline | α-CH_2_ |
| 4.12 | q | Lactate | α-CH |
| 4.15 | t | Pro | α-CH |
| 4.26 | m | Thr | β-CH |
| 4.34 |  | ATP |  |
| 4.65 | d | β-Glucose | C1H |
| 5.2 | d | Trehalose | C1H |
| 5.24 | d | α-Glc | C1H |
| 5.42 | d | Sucrose | G1H |
| 5.94 | d | GTP | C1'H ribose |
| 6.11 | d | Inosine | C1'H ribose |
| 6.15 | d | ATP | C1'H ribose |
| 6.52 | s | Fumarate |  |
| 6.9 | d | Tyr | C3, 5H ring |
| 7.12 | s | His | C4H ring |
| 7.21 | d | Tyr | C2, 6H ring |
| 7.22 | t | Trp | C5H ring |
| 7.29 | t | Trp | C6H ring |
| 7.33 | d | Phe | C2, 6H ring |
| 7.4 | m | Phe | C4H ring |
| 7.44 | m | Phe | C3, 5H ring |
| 7.54 | d | Trp | C7H ring |
| 7.73 | d | Trp | C4H ring |
| 7.94 | s | His | C2H ring |
| 8.15 | s | GTP | C8H ring |
| 8.19 | s | Inosine | C8H ring |
| 8.27 | s | ATP | C2H ring |
| 8.35 | s | Inosine | C2H ring |
| 8.46 | s | Formate |  |
| 8.55 | s | ATP | NH ring |

s, singlet; d, doublet; t, triplet; q, quartet; m, multiplet; dd, doublet of doublet.

**Table SII**. List of the amino acids identified by the EZ:faast Free (Physiological) Amino Acid kit in *C. elegans*.

| ***RT (min)*** | ***Metabolite*** |
| --- | --- |
| 1.29 | Alanine |
| 1.39 | Glycine |
| 1.49 | α-aminobutyric acid |
| 1.60 | Valine |
| 1.73 | Norvaline (IS) |
| 1.82 | Leucine |
| 1.88 | Isoleucine |
| 2.10 | Threonine |
| 2.20 | Proline |
| 2.30 | Asparagine |
| 2.66 | Thiaproline |
| 2.87 | Aspartic acid |
| 2.90 | Methionine |
| 3.24 | Glutamic acid |
| 3.27 | Phenylalanine |
| 3.55 | α-aminoadipic acid |
| 3.90 | Glutamine |
| 4.30 | Ornithine |
| 4.58 | Lysine |
| 4.77 | Histidine |
| 5.06 | Tyrosine |
| 5.35 | Tryptophan |
| 5.85 | Cystathionine |

**Table SIII.** Characteristics of the PLS-DA models obtained considering each strains against the wild type in the GC-MS experiments using the EZ:faast Free (Pysiological) Amino Acid kit.

|  | *n LV* | *R^2^(X)* | *R^2^(Y)* | *Q^2^* |
| --- | --- | --- | --- | --- |
| **WT vs *fat-5*** | 3 | 0.614 | 0.989 | 0.878 |
| **WT vs *fat-6*** | 4 | 0.892 | 0.988 | 0.903 |
| **WT vs *fat-7*** | 3 | 0.668 | 0.988 | 0.932 |
| **WT vs *fat-5;fat-7*** | 2 | 0.587 | 0.98 | 0.934 |
| **WT vs *fat-5;fat-6*** | 3 | 0.815 | 0.984 | 0.932 |

**Table SIV**. List of the most significant amino acids changes considering each mutant against the wild type.

|  | *High Positive Loading Values* | *High Negative Loading Values* |
| --- | --- | --- |
| **WT (positive score values) vs *fat-5* (negative score values)** | Alanine Isoleucine  Valine Cystathionine  Proline Histidine  α-aminoadipic acid | Phenylalanine Tyrosine  Glutamic acid Methionine  Aspartic acid Asparagine  Lysine |
| **WT (positive score values) vs *fat-6* (negative score values)** | Threonine  Glutamic acid  Thiaproline Cystathionine  Aspartic acid | α-Aminobutyric acid Ornithine  Glycine Alanine  α-aminoadipic acid  Glutamine |
| **WT (positive score values) vs *fat-7* (negative score values)** | Tyrosine Leucine  Isoleucine Threonine  Valine Thiaproline  Proline Methionine  Phenylalanine | Glycine Ornithine  α-Amino butyric acid Alanine  Cystathionine Glutamine  α-aminoadipic acid |
| **WT (positive score values) vs *fat-5;fat-7* (negative score values)** | Leucine Isoleucine  Phenylalanine Proline  Threonine Methionine  Glutamic acid Tyrosine | Ornithine Glutamine  Glycine  α–Aminobutyric acid  Histidine Alanine α-aminoadipic acid |
| **WT (positive score values) vs *fat-5;fat-6* (negative score values)** | Glutamine Isoleucine  Methionine Valine  Glutamic acid Threonine  Aspartic acid  α-aminoadipic acid | Histidine Lysine  Phenylalanine Tyrosine  Ornithine Asparagine  Tryptophan |

**Table SV**. List of the fatty acids identified by the GC-FID analysis of *C. elegans*.

| **RT (min)** | **Metabolite** |
| --- | --- |
| 9.77 | Lauric acid methyl ester C12:0 |
| 10.66 | D-25 tridecanoic acid methyl ester C13:0 |
| 12.45 | Myristic acid methyl ester C14:0 |
| 13.34 | 13-methyl tetradecanoic acid methyl ester C15:0 ISO |
| 13.58 | 12-methyl tetradecanoic acid methyl ester C15:0 ANTEISO |
| 14.19 | Pentadecanoic acid methyl ester C15:0 |
| 15.21 | 14-methyl pentadecanoic acid methyl ester C16:0 ISO |
| 16.19 | Palmitic acid methyl ester C16:0 |
| 16.70 | Palmitoleic acid methyl ester C16:1 |
| 17.31 | 15-methyl tetradecanoic acid methyl ester C17:0 ISO |
| 18.37 | Margaric acid methyl ester C17:0 |
| 18.98 | cis 9,10-methylene hexadecanoic acid methyl ester C17:0Δ |
| 20.70 | Stearic acid methyl ester C18:0 |
| 21.13 | Oleic acid methyl ester C18:1n9c |
| 21.31 | Vaccenic acid methyl ester C18:1n7c |
| 22.15 | 9,12-Octadecadienoic acid methyl ester C18:2n6 |
| 22.84 | 6,9,12-Octadecatrienoic acid methyl ester C18:3n6 |
| 23.10 | cis 9,10-methylene octadecanoic acid methyl ester C19:0Δ |
| 23.68 | 9,12,15-Octadecatrienoic acid methyl ester C18:3n3 |
| 25.49 | Arachidic acid methyl ester C20:0 |
| 25.92 | 11-Eicosenoic acid methyl ester C20:1 |
| 27.01 | 11,14-Eicosadienoic acid methyl ester C20:2n6 |
| 27.62 | 8,11,14-Eicosatrienoic acid methyl ester C20:3n6 |
| 28.13 | Arachidonic acid methyl ester C20:4n6 |
| 28.55 | 11,14,17-Eicosatrienoic acid methyl ester C20:3n3 |
| 29.17 | 8,11,14,17-Eicosatetraenoic acid methyl ester C20:4n3 |
| 29.70 | 5,8,11,14,17-Eicosapentenoic acid methyl ester C20:5n3 |

**Table SVI.** Characteristics of the PLS-DA models obtained considering each strains against the wild type in the GC-MS experiments of the lipid fraction.

|  | *n LV* | *R^2^(X)* | *R^2^(Y)* | *Q^2^* |
| --- | --- | --- | --- | --- |
| **WT vs *fat-5*** | 2 | 0.773 | 0.927 | 0.831 |
| **WT vs *fat-6*** | 2 | 0.53 | 0.907 | 0.803 |
| **WT vs *fat-7*** | 2 | 0.498 | 0.966 | 0.838 |
| **WT vs *fat-5;fat-7*** | 3 | 0.619 | 0.991 | 0.927 |
| **Wt vs *fat-5;fat-6*** | 2 | 0.592 | 0.959 | 0.741 |

**Table SVII**. List of the most significant fatty acid changes considering each mutant against the wild type.

|  | *High Positive Loading Values* | *High Negative Loading Values* |
| --- | --- | --- |
| **WT (positive score values) vs *fat-5* (negative score values)** | C18:2n6 C17:0 ISO  C20:4n6 C20:1n9  C15:0 ISO C16:1n9  C18:1n7 C20:5n3  C18:1n9 C20:3n6  C18:3n6 C20:4n3 | C14:0  C18:0  C16:0  C21:0  C15:0 ANTEISO |
| **WT (positive score values) vs *fat-6* (negative score values)** | C20:4n3 C18:2  C20:3n6 C20:5n3  C18:3n6 C18:1n9  C15:0 ISO C17:0 ISO | C18:0 C16:0  C14:0 C20:3n3  C20:0  C17:0 DELTA |
| **WT (positive score values) vs *fat-7* (negative score values)** | C20:4n3 C20:3n6  C18:1n9 C20:5n3  C18:3n6 C15:0 ISO  C17:0 ISO  C15:0 ANTEISO  C18:2n6 C16:1 | C18:0  C16:0  C14:0  C20:3n3  C15:0 |
| **WT (positive score values) vs *fat-5;fat-7* (negative score values)** | C16:1 C20:1n9  C20:5n3 C18:1n7  C15:0 ISO C17:0 ISO  C18:3n3 C16:0 ISO | C17:0 C16:0  C18:0 C15:0  C18:3n6 C20:3n3  C18:1n9 |
| **WT (positive score values) vs *fat-5;fat-6* (negative score values)** | C18:2n6 C16:1  C15:0 ISO C17:0 ISO  C20:1 C20:4n3  C18:1n9 C18:2n6  C20:4n6 C20:5n3  C20:3n6 | C15:0 ANTEISO  C20:3n3  C18:0  C16:0  C14:0 |

**Table SVIII.** Characteristics of each PLS-DA models obtained considering each strains against the wild type in the LC-MS experiments of the lipid fraction.

|  | *n LV* | *R^2^(X)* | *R^2^(Y)* | *Q^2^* |
| --- | --- | --- | --- | --- |
| **WT vs *fat-5*** | 1 | 0.35 | 0.915 | 0.854 |
| **WT vs *fat-6*** | 3 | 0.494 | 0.992 | 0.895 |
| **WT vs *fat-7*** | 4 | 0.551 | 0.996 | 0.789 |
| **WT vs *fat-5;fat-7*** | 2 | 0.428 | 0.989 | 0.938 |
| **WT vs *fat-5;fat-6*** | 2 | 0.355 | 0.973 | 0.791 |
